# IoT Application of Transfer Learning in Hybrid Artificial Intelligence Systems for Acute Lymphoblastic Leukemia Classification

**DOI:** 10.3390/s21238025

**Published:** 2021-12-01

**Authors:** Krzysztof Pałczyński, Sandra Śmigiel, Marta Gackowska, Damian Ledziński, Sławomir Bujnowski, Zbigniew Lutowski

**Affiliations:** 1 Faculty of Telecommunications, Computer Science and Electrical Engineering, Bydgoszcz University of Science and Technology, 85-796 Bydgoszcz, Poland; krzysztof@palczynski.com.pl (K.P.); marta.gackowska@pbs.edu.pl (M.G.); damian.ledzinski@pbs.edu.pl (D.L.); slawomir.bujnowski@pbs.edu.pl (S.B.); zbigniew.lutowski@pbs.edu.pl (Z.L.); 2 Faculty of Mechanical Engineering, Bydgoszcz University of Science and Technology, 85-796 Bydgoszcz, Poland

**Keywords:** hybrid artificial intelligence system, MobileNet v2, IoT, low-resource dataset, lymphocyte cells, leukemia, ALL-IDB database

## Abstract

Acute lymphoblastic leukemia is the most common cancer in children, and its diagnosis mainly includes microscopic blood tests of the bone marrow. Therefore, there is a need for a correct classification of white blood cells. The approach developed in this article is based on an optimized and small IoT-friendly neural network architecture. The application of learning transfer in hybrid artificial intelligence systems is offered. The hybrid system consisted of a MobileNet v2 encoder pre-trained on the ImageNet dataset and machine learning algorithms performing the role of the head. These were the XGBoost, Random Forest, and Decision Tree algorithms. In this work, the average accuracy was over 90%, reaching 97.4%. This work proves that using hybrid artificial intelligence systems for tasks with a low computational complexity of the processing units demonstrates a high classification accuracy. The methods used in this study, confirmed by the promising results, can be an effective tool in diagnosing other blood diseases, facilitating the work of a network of medical institutions to carry out the correct treatment schedule.

## 1. Introduction

Acute lymphoblastic leukemia (ALL) comprises a group of lymphoid neoplasms that morphologically and immunophenotypically resemble B- and T-lineage precursor cells. ALL is the most common neoplasm in children, with a peak incidence between the ages of 2 and 5 years [1], whereas it is scarce in older patients (over 60 years of age) [2]. Diagnosis includes mainly a microscopic examination of blood and bone marrow [3].

The diagnosis of leukemias, including acute myeloid leukemia, requires standardized methods of classification. One of the most commonly used and oldest methods is the French-American-British (FAB) morphological classification [4]. For lymphoblastic leukemia, based on cytological features and the degree of heterogeneity in the distribution of leukemic cells according to the FAB classification, the following types are distinguished: L1, L2, and L3. The characteristics of type L1 are the predominance of small cells, homogeneous nuclear chromatin, and a regular nucleus shape with possible cleavages present. The nuclei are invisible or small and inconspicuous; the amount of cytoplasm is scanty; and the cytoplasmic vacuolization is variable. Deep cytoplasmic basophilia is uncommon.

The L2 type is characterized by a large and heterogeneous cell size and variable heterogeneous nuclear chromatin in each case. The shape of the nucleus is irregular, and the nucleolus is at least one and is often large. The amount of cytoplasm is variable but is often moderately abundant. The cytoplasmic vacuolization is variable.

The characteristics of the L3 type are a large and uniform cell shape and a finely spotted and uniform chromatin of the nucleus. The nucleus has a regular oval–round shape. Nucleoli are prominent and one or more are vesicular. The amount of cytoplasm is moderately abundant, and the basophilia of the cytoplasm is intense. The challenge in the correct classification is that, as described above, blood cells differ from one another in terms of cytological features and the degree of heterogeneity in their distribution. The features that allow the differentiation of malignant cells and the type of the disease are, among others: the amount of cytoplasm, cell vacuolization, and the shape and size of the cell nucleus or nucleolus. The traditional method is to manually analyze the differences and observe the cells under an electron microscope by an experienced physician. Correct manual classification requires both experience and specialist knowledge. Therefore, there may be some differences between the results obtained. Thanks to artificial intelligence methods, it is possible to speed up the work of medics and increase the effectiveness and repeatability of results.

Artificial intelligence is a widely discussed issue in the world of science and technology for solving engineering problems. However, it is essential to realize that recent research in this area presents advanced applications of artificial intelligence in fields other than medicine, including computer science for developing new methods and algorithms and [5,6] in petroleum engineering [7] or even in civil engineering [8]. In this study, a hybrid artificial intelligence solution was used in medicine, and, at the same time, it is a promising method that can be used in IoT networks.

Some studies focus on image-segmentation methods to locate white blood cells on a microscopic image. In [9], input images were converted from RGB color space to haematoxylin-eosin-DAB (HED) space. Then bilateral filter and canny edge segmentation were used to extract individual lymphocytes. A watershed algorithm was finally used to determine the seed of each region. This method showed an accuracy of over 90%, with low computational complexity and execution time. In turn, the work [10] used the conversion of RGB to CMYK and L * a * b and the clustering algorithm K-means; in post-processing, dilation and erosion were used. The results obtained in the experiments had a Kappa index of 0.9306 in the ALL-IDB 2, 0.8603 in the BloodSeg, and 0.9119 in the leukocytes database.

However, the main challenge in diagnosing the disease is the correct classification of malignant lymphocytes. The study [11] proposed the architecture of deep neural networks using the AlexNet model from CNN, and it used softmax to classify acute lymphoblastic leukemia into its subtypes and normal state. The method used also included transfer learning. The segmentation approach based on the simple threshold method was used to prepare the data to distinguish the region of interest. For the developed method and the test set of 330, the accuracy was 97.78%. In turn, the authors in [12], apart from AlexNet, used ImageNet, and, for 33 images from the ALL-IDB database, the system correctly identified 94.1% of lymphoblasts. Convolutional neural network ResNeXt50 with squeeze-and-excitation modules was used in [13] to classify ALL. Initially, the network was pre-trained on ImageNet. An accuracy of 89.7% was achieved.

On the other hand, the authors of the work [14] used the convolutional neural network to classify types of leukemia, such as AML, CLL, CML, and ALL, and healthy patients. In addition, data augmentation was used to diversify the data set. As a result, an 81% efficiency was achieved with 231 test samples in classifying all leukemia subtypes. In addition, cross-validation was used in all experiments.

The authors [15] proposed the Siamese network-based few-shot learning method to classify leukocytes. The Siamese network described in the work contains two convolutional neural subnets with the same structure to know the vector of input images and to share weights. In addition, a two-way one-shot support set was used, which was used as additional information supporting the classification. The average accuracy of the classification of basophil and eosinophil cells using the Siamese network was 89.66.

The work [16] describes the automatic classification of leukocytes. This method can be divided into three main parts. Initially, white blood cells (WBC) are isolated from the microscopic examination of blood using R–B conversion, threshold segmentation, and binarization. These include eosinophils, basophils, neutrophils, monocytes, and lymphocytes. The PRICoLBP function was then used to reflect the granularity of eosinophils and basophils, which increased their discriminatory power with other WBC types. Then, the stage for which convolutional neural networks were used was the isolation of the constants of three kinds of WBC: neutrophils, monocytes, and lymphocytes. CNN is a special feedforward neural network that consists of several convolutional layers and pooling layers. Finally, with the help of the Random Forest algorithm, the three remaining WBCs were classified. The developed method allowed for an average detection accuracy of 92.8%, while the lowest accuracy was demonstrated when recognizing lymphocytes. In [17], the green component/channel of the RGB microscopic image (input image) was extracted at the beginning. Then, the threshold segmentation, the opening operator, and the border-cleaning techniques were applied to the obtained binary image. Next, the bounding box technique was used to trim each WBC to a single image, while the cosine transform extracted textures and features. Finally, kNN, SVM, and naïve Bayes were used to segregate normal and abnormal cells. The classification of the disease was 97.45%.

This study focused on researching the application of transfer learning by using publicly available pre-trained neural networks as a significant part of hybrid artificial intelligence systems to offset the shortage of domain-specific data and low computational capabilities. In this work, the MobileNet v2 [18] network pre-trained on the ImageNet dataset was used for encoding images into small feature vectors, making them processable for CPU-friendly machine learning models like XGBoost [19] or Random Forest [20]. The MobileNet v2 architecture was employed because it was optimized for small processing units like mobile CPUs or IoT. The results were compared with the bare MobileNet v2 network repurposed for this task and the designed convolutional neural network as a baseline for comparison. Due to the use of advanced artificial intelligence solutions, it was possible to correctly classify and differentiate the disease and the correct state in the diagnosis of ALL. This could effectively diagnose their blood diseases and facilitate a network of medical facilities to undertake the proper treatment schedule. A novelty in this article was the use of hybrid artificial intelligence. The neural network was pre-trained on a vast dataset and then encoded data in a specific set. Then, machine learning models were trained on coded and reduced data. This gave a great advantage for use in IoT networks while demonstrating high classification accuracy.

This article is organized as follows. Section 2 closely describes the methods, the architectures of the hybrid artificial intelligence system, and the previously carried-out image processing. Then, Section 3 presents the results of the research. Then, the discussion is given in Section 4. Finally, Section 5 concludes the article and provides a look at further studies on this topic.

## 2. Materials and Methods

**Figure 1:**
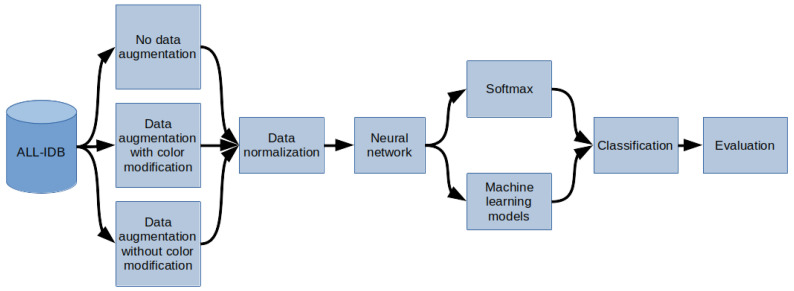
General overview diagram of the method.

The methodology of the research described in this paper is depicted in Figure 1. In the first step, the data from the ALL-IDB database were sampled. Then, depending on the model of the experiment, sampled images were either preprocessed using augmentation or left intact. Depending on the selected experiment options, the color modification was not applied during the augmentation process to test the impact of timbre change on overfitting prevention. In the next step, regardless of whether augmentation was used, the z-score normalization was performed into images into the work characteristic of the MobileNet v2 network. At the end of this step, data preprocessing was finished, and the images were ready to be interpreted by the artificial intelligence system.

The prepared data entered the system by being processed by a neural network, serving as an encoder, to extract feature vectors from the input images. Then, data encoded in feature vectors are passed to the classification module, which is explained further in the article as the “head” for classification. The “head” module is either a one-layer fully-connected neural network or one of three machine learning models: XGBoost, Random Forest, or Decision Tree. This process is described in detail in the section “Hybrid Artificial Intelligence system.” Finally, in the last step, the results of the artificial intelligence system are evaluated.

### 2.1. ALL-IDB Database

**Figure 2:**
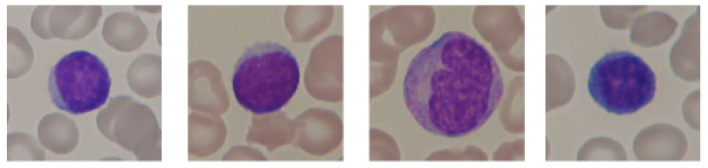
An example of segmented lymphocytes belonging to the non-leukemia class.

**Figure 3:**
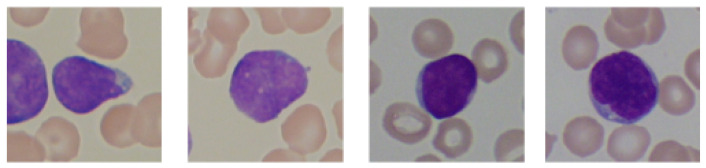
An example of segmented lymphocytes belonging to the leukemia class.

The study used images of lymphocyte cells, healthy patients, and patients with acute lymphocytic leukemia. The dataset was an ALL-IDB dataset, which was downloaded with the owner’s consent [17,21,22,23]. The ALL-IDB dataset is a public dataset of microscopic images of peripheral blood cells that have been developed for segmentation, evaluation, and classification. The data contained in the database are considered reliable, as oncologists annotate them. The ALL-IDB database has two distinct versions (ALL-IDB1 and ALL-IDB2). In this study, experiments were performed on images from ALL-IDB2. ALL-IDB2 is a set of excised regions of interest from blood-smear images taken from healthy patients and leukemia patients, who belong to the ALL-IDB1 dataset. The ALL-IDB2 dataset is a subset of 260 segmented images, with 50% containing normal leukocytes and the remaining containing malignant cells (Figure 2 and Figure 3).

### 2.2. Image Preprocessing

The following data-augmentation techniques were used to increase the size of the training set:

- color jitter,
- Gaussian blur,
- horizontal flip,
- vertical flip,
- rotation.

**Figure 4:**
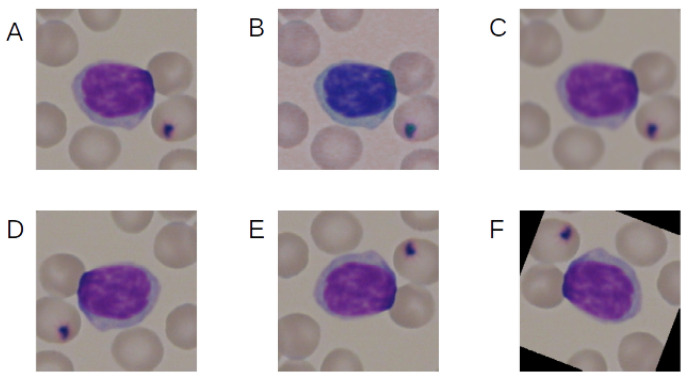
Example of the effect of the augmentation techniques used. (**A**) No augmentation, (**B**) color jitter, (**C**) Gaussian blur, (**D**) horizontal flip, (**E**) vertical flip, and (**F**) rotation.

Figure 4 shows the example of the effect of the augmentation techniques used.

Two variants of augmentation were used, and color jitter was used only in one of them. After augmentation, all images from the database had been normalized. It consisted of subtracting the means, which were 0.485, 0.456, and 0.406, and dividing by the standard deviations, which were 0.229, 0.224, and 0.225.

### 2.3. Hybrid Artificial Intelligence System

**Figure 5:**
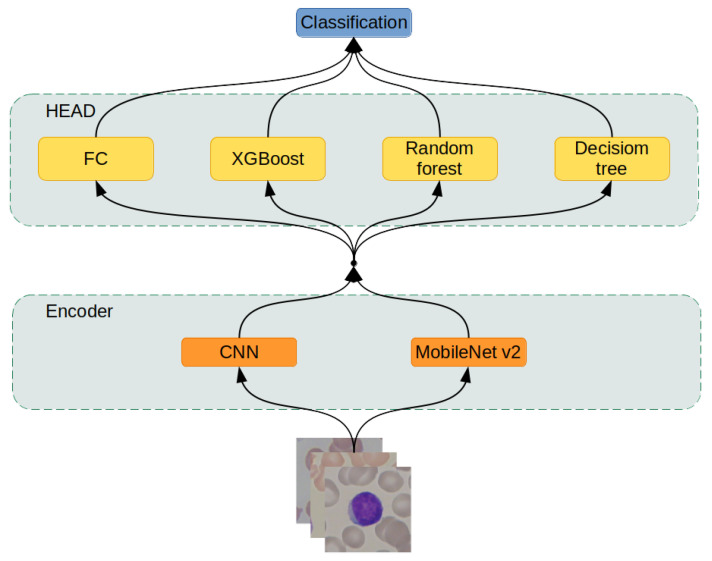
Hybrid artificial intelligence system architecture.

This research designed artificial intelligence models from two modules: the encoder and the head (Figure 5). The encoder transforms the input image into a fixed-size feature vector, abstractly describing the input data. The head takes the feature vector computed by the encoder and performs the classification; such a division of responsibilities allowed for the modular structure of the artificial intelligence system. Two neural networks were used as encoders and four different machine learning models as heads.

#### 2.3.1. Encoders

The first is the primary deep convolutional neural network with the standard, homogeneous architecture described in Table 1. This network is referred to as the CNN-Encoder further in the article. It was designed without significant structural improvements like residual connections [24] or inception-based [25] layers to serve as a baseline for comparison during model evaluation.

The output value was flattened into a 128-dimensional vector. After each convolutional layer, the Leaky ReLU activation function was applied with a negative slope coefficient α=0.01. Each convolutional layer with kernel 3×3 had a padding value to offset stride and to preserve activation maps’ dimensionality. The max pooling layers performed the shrinkage of the above-mentioned activation maps. The last layer performed convolution by applying kernel 1×1 to reduce the number of channels from 128 to 2, resulting in the final encoded feature vector size being reduced by the factor of 64.

The second network used as an encoder was MobileNet V2. This network was selected due to its trade-off between performance and efficiency on mobile CPUs. The MobileNet architecture is more complex than the first one due to the usage of numerous structural improvements like:

- Depthwise separable convolutions—which improves the convolutional layers. In a normal convolutional layer, the equation gives a convolutional layer kernel that has size (w,h,d) where *w* (width) and *h* (height) are arbitrarily chosen hyperparameters and *d* (depth) is equal to the depth of the input tensor. As a result, the amount of weights required to be optimized to train the *i*-th convolutional layer equation is given by the following equation:
  (1) $$|\Theta_{i}|=n_{i}\cdot w_{i}\cdot h_{i}\cdot d_{i}$$
  where $n_{i}$ is the number of filters in a layer. This relationship between the input tensor’s depth and the number of weights in one filter becomes cumbersome during the stacking of deep convolutional layers. For example, the convolutional layer gets an input tensor of depth 1024 and must preserve the size in the third dimension. These restrictions imply that $d_{i}=1024$ and the number of filters is also $1024\cdot d_{i}\cdot w_{i}\cdot h_{i}=1,048,576\cdot w_{i}\cdot h_{i}$. Since $w_{i},h_{i}\in N^{+}$—0, 1, 2, it means that the minimum amount of weights required is equal to 9,437,184. This amount of weights is staggering, taking into consideration that it is merely one convolutional layer. Depthwise separable convolutions reduce this problem by splitting the convolutional layer into two parts: the first one applies one filter of kernel $w_{i}\cdot h_{i}$ without depth to every channel instead of having filters interpreting every channel, and the second layer uses a 1×1 convolution on the output of the first layer, performing a depth-sensitive linear transformation. As a result, the same task is completed, but the equation gives the cost of the weights:
  (2) $$|\Theta_{i}^{\prime }|=n_{i}\cdot w_{i}\cdot h_{i}+n_{i}\cdot d_{i}\cdot 1\cdot 1=n_{i}\cdot \left(w_{i}\cdot h_{i}+d_{i}\right)$$
  Since $n_{i}=d_{i}$ and usually $w_{i},h_{i}<<d_{i}$, the maximum reduction obtained from using this method is equal to
  (3) $$\lim_{n_{i}\to \infty }\frac{n_{i}^{2}+n_{i}w_{i}h_{i}}{n_{i}^{2}w_{i}h_{i}}=\frac{1}{w_{i}h_{i}}\left(1+\lim_{n_{i}\to \infty }\frac{w_{i}h_{i}}{n_{i}}\right)=\frac{1}{w_{i}h_{i}}\left(1+0\right)=\frac{1}{w_{i}h_{i}}$$
  As a result, this method is able to reduce the number of used weights by the factor of $w_{i}\cdot h_{i}$.
- Linear bottlenecks—a newly introduced layer performs a linear transformation of the convolutional layers’ activation map, resulting in tensor’s depth reduction with minimal information loss and an increasing amount of information stored per channel.
- Inverted residuals—the residual connection between layers bottlenecks instead of connecting normal convolutional layers. Since bottleneck layers are by design depth-reduced transformations of convolutional layers, application of a residual connection by bundling bottleneck layers results in a further computation reduction.

In this research, MobileNet v2 was used in two different versions: pretrained and not pretrained. The pretrained network was optimized to solve tasks from the ImageNet [26] contest consisting of image classification into a thousand different classes. The not-pretrained network started training using weights initialized by the usage of the Kamming He initialization algorithm.

#### 2.3.2. Head

The head module takes as an input a feature vector computed by the encoder and performs its classification. There were four modules chosen for this task:

- Fully connected neural network layer,
- XGBoost,
- Random Forest,
- Decision Tree [27].

The first module is part of the neural networks, and it is able to propagate error gradients further down the network. Because of that, this particular head can be trained together with the encoder. However, the rest of the modules require a fully trained encoder to encode images into small feature vectors.

The hybrid approach allows the network already pre-trained on different tasks (like ImageNet) and uses it as a finished encoder to create a new dataset to translate the original one into the feature space. Then, machine learning algorithms can be trained on the newly created dataset of feature vectors, utilizing their different approach to create a heterogeneous classification system.

### 2.4. Training

In this research, two different training techniques for neural-network-only systems and hybrid machine learning models were used. In both of these methods, data augmentation was used in one of three modes:

- no augmentation was applied,
- augmentation was applied with all of the available techniques described in the “Image preprocessing” section,
- augmentation was applied with all the techniques except the “Color jitter” method.

The training was performed using the following hardware configurations: dual-Intel Xeon Silver 4210R, 192 GB RAM, and Nvidia Tesla A100 GPU. In this research, PyTorch, Sklearn, Numpy, Pandas, and Jupyter Lab programming solutions were used to implement the neural networks [28].

#### 2.4.1. Neural Network Training

This procedure was employed when a fully connected layer was used as a head of the system. Because all elements in the system can propagate gradient error, both the encoder and the head were trained simultaneously. The models were trained using this procedure:

- CNN-Encoder + fully connected layer,
- not-pretrained MobileNet v2 + fully connected layer,
- pretrained MobileNet v2 + dully connected layer.

Neural networks were trained using the Adam optimizer [29]. Every network was optimized on a training dataset and evaluated on a validation dataset. They were trained for 1000 epochs unless early stopping [30] was performed. If the best result on the validation dataset was not improved in 100 epochs, training was stopped, and another network was created. The learning rate at the beginning was equal to 0.001, and it was reduced by half if the network did not improve its best result on the training dataset within 10 epochs from the last improvement or learning rate reduction. If the learning rate reached 0.000001, then no further reduction was applied. Depending on the augmentation settings selected, each image might be subjected to random augmentation before being put on the input of the neural network.

#### 2.4.2. Hybrid System Training

The hybrid system consisted of a MobileNet v2 encoder pre-trained on the ImageNet dataset and a machine learning algorithm performing the role of the head. These algorithms were:

- XGBoost,
- Random Forest,
- Decision Tree.

To train these head models, a new dataset of encoded images was created. Thus, there were three datasets created. The first one was not subjected to augmentation. In this case, every image in the original dataset was converted into a 1000-dimensional feature vector.

In both the second and the third cases, augmentation was used, resulting in every image being 100 times randomly augmented and its vector added to the dataset. As a result, datasets with an applied boost had a size 100 times greater than the non-augmented one. The difference between the second and third cases was in whether color jitter was used or not.

After datasets creation, each machine learning model was optimized on this set according to its unique training algorithm.

### 2.5. Metrics

Neural networks were evaluated using the metrics described below [28]. For the purpose of simplicity of equations, certain acronyms were created, as follows: TP—true positive, TN—true negative, FP—false positive, and FN—false negative. The metrics used for network evaluation were:

- Accuracy: Acc=(TP+TN)/(TP+FP+TN+FN),
- Precision=TP/(TP+FP),
- Recall=TP/(TP+FN),
- F1=2∗Precision∗Recall/(Precision+Recall),
- AUC—area under the receiver operating characteristic (ROC). The ROC is a curve determined by calculating the true-positive rate = TFP=TP/(TP+FN) and the false-positive rate = FPR=FP/(TN+FP). The false-positive rate describes the x-axis and the true-positive rate the y-axis of a coordinate system. By changing the threshold value responsible for classification of an example as belonging to either the positive or negative class, pairs of TFP-FPR were generated, resulting in the creation of the ROC curve. The AUC is a measurement of the area below the ROC curve.

## 3. Results

Training, validation, and test sets were generated 15 times to evaluate networks to minimize the influence of random dataset division. Each network was trained on a training dataset. During the training, the network was assessed on the validation dataset to select the best, least-overfitted weights set of the network, and to perform early stopping. When such a set of weights was established, the final network’s evaluation was performed on the test dataset. Results of the networks were grouped by both architecture selection, whether pre-training was employed or not, application of augmentation, and presence of color modification during the augmentation process. The results are presented in Table 2.

## 4. Discussion

The three best results were obtained from the pre-trained MobileNet v2 repurposed to blast cell detection through learning with a fully connected layer head attached. However, MobileNet v2, not pre-trained with a fully connected layer head, scored much lower despite having the same architecture. It suggests that transfer learning can be used as a regularization technique, preventing overfitting and improving overall performance. In addition, ImageNet contained images depicting objects and animals related to everyday daily life instead of pictures of microbiological phenomena, yet such pre-training proved beneficial. It further suggests that a pre-training network on large datasets from seemingly unrelated domains may improve the results on small, specialized tasks like blast-cell classification in this particular research.

Hybrid systems with MobileNet v2 as an encoder scored the best after the repurposed, pre-trained MobileNet v2 network. Their performance was better than the CNN-Encoder network and the not-pre-trained MobileNet. It suggests that in case of limited access to high-end processing units like GPUs, the strategy described below may have satisfactory results:

- take the available neural network pre-trained on a massive dataset,
- use this network to encode data in the small, domain-specific dataset,
- train a machine learning model on reduced, encoded data.

This strategy may be performed on the CPU. The computational bottleneck in this operation is using a deep neural network on the CPU to encode the dataset. However, this operation must be conducted only once. Its result is sufficient for machine learning model training and is much lighter than the original dataset, making it easier to store. In this research, both machine learning models like XGBoost and the MobileNet v2 network as an encoder were evaluated due to their proficiency in training on the CPU. Such an approach may prove beneficial for systems with reduced computational capabilities like mobile devices or the IoT.

The XGBoost and Random Forest algorithms proved to be capable of extracting abstract information from encoded feature vectors. However, the Decision Tree algorithm scored substantially worse and did not achieve the desired results. Moreover, this algorithm’s simplistic structure was not complex enough to extract the high-level information required for performing classification on a sparse dataset like the one examined in this work.

The pre-trained MobileNet v2 scored better results than CNN-Encoder, which in turn scored better than the not-pre-trained MobileNet v2. These findings suggest that MobileNet v2 was not pre-trained overfitted to the training set due to its more profound and more complicated structure compared with the baseline CNN-Encoder. However, pre-training allowed MobileNet v2 to score better than CNN-Encoder. Thus, it suggests that designing networks seemingly more profound than required and pre-training them may provide better results than applying smaller architectures despite the concern of overfitting an overparameterized model.

The augmentation mode was split into augmentation with the application of color jitter and without it. Because of an a priori assumption, color was an essential factor in cell classification as it is an indicator of biological features. This assumption proved to be correct because top architectures differentiating between themselves only by applying color jitter scored better without this augmentation technique. This proves that augmentation techniques must be challenged to determine whether they are truly beneficial or not for this particular dataset’s purposes.

The pre-trained MobileNet v2 network proved its effectiveness in the researched problem despite the training process being conducted on data from different domains. It suggests that the domains of knowledge are not as separated as it seems. However, it is doubtful that understanding the MobileNet v2 network gathered during training on the ImageNet dataset is helpful in this example. It is presumed that only a specific part of this network is useful in this topic. The procedure for such knowledge extraction would be beneficial as it reduces the computation and size of stored weights sets. The authors plan on further investigation of this topic.

## 5. Conclusions

The proposed strategy of designing hybrid artificial intelligence systems for low-resource, low-computational-complexity processing units’ tasks by introducing a pre-trained neural network for data encoding proved beneficial in this particular task. The examined systems using MobileNet v2 as an encoder and XGBoost and Random Forest as classification heads were able to score, on average, an above 90% accuracy, going as high as 97.4%. The system developed in this work can be trained and run on a low-power CPU like a mobile CPU or one dedicated to the IoT. However, the Decision Tree algorithm turned out to be not complex enough to perform meaningful classification. The best results were obtained by repurposing the already trained deep neural network instead of training the same one from scratch or creating the smaller one to reduce overfitting. The regularization benefit of transfer learning was significant during the examination of this dataset.

## Figures and Tables

**Table 1:** Architecture of the deep convolutional neural network.

Layer	Channels In	Channels Out	Kernel Size	Padding	Stride
Conv2d	3	8	3 × 3	1 × 1	1 × 1
MaxPool2d	8	8	3 × 3	0 × 0	3 × 3
Conv2d	8	16	3 × 3	1 × 1	1 × 1
MaxPool2d	16	16	3 × 3	0 × 0	3 × 3
Conv2d	16	32	3 × 3	1 × 1	1 × 1
MaxPool2d	32	32	3 × 3	0 × 0	3 × 3
Conv2d	32	64	3 × 3	1 × 1	1 × 1
MaxPool2d	64	64	3 × 3	0 × 0	3 × 3
Conv2d	64	128	3 × 3	1 × 1	1 × 1
MaxPool2d	128	128	3 × 3	0 × 0	3 × 3
Conv2d	128	2	1 × 1	0 × 0	1 × 1

**Table 2:** Experiment results

Name	Acc	Acc Avg|Std	F1	F1 Avg|Std	AUC	AUC Avg|Std
FC, Mobilenet v2, augmented with no color	100.0–82.0%	94.8% | 5.3	100.0–81.8	94.8 | 5.3	100.0–95.5	99.2 | 1.3
FC, Mobilenet v2, augmented	100.0–87.1%	93.8% | 3.8	100.0–86.8	93.7 | 3.9	100.0–93.7	99.0 | 1.6
FC, Mobilenet v2, no augmentation	100.0–76.9%	92.8% | 6.1	100.0–76.8	92.7 | 6.1	100.0–94.1	98.7 | 1.6
Random Forest, Mobilenet v2 augmented	97.4–84.6%	92.1% | 4.0	97.4–84.5	92.0 | 4.1	100.0–95.6	98.7 | 1.3
XGBoost, Mobilenet v2, augmented with no color	97.4–82.0%	91.1% | 5.1	97.4–81.2	90.9 | 5.2	100.0–93.2	97.8 | 2.2
XGBoost, Mobilenet v2, augmented	97.4–76.9%	91.1% | 5.5	97.4–76.8	91.0 | 5.5	100.0–89.7	98.0 | 2.8
Random Forest, Mobilenet v2, augmented with no color	94.8–82.0%	89.9% | 4.3	94.8–81.6	89.8 | 4.4	99.7–92.8	97.9 | 1.9
Decision Tree, Mobilenet v2, augmented	89.7–64.1%	80.0% | 7.7	89.5–63.8	79.7 | 7.8	89.5–63.9	80.1 | 8.0
Decision Tree, Mobilenet v2, augmented with no color	89.7–66.6%	79.3% | 6.6	89.6–65.8	79.0 | 6.8	90.3–67.7	79.8 | 6.6
Random Forest, Mobilenet v2, no augmentation	87.1–64.1%	76.9% | 6.9	87.1–63.8	76.7 | 7.0	94.8–75.6	85.2 | 5.4
XGBoost, Mobilenet v2, no augmentation	89.7–56.4%	75.3% | 11.6	89.7–55.9	75.1 | 11.7	95.7–65.7	83.0 | 9.4
Decision Tree, Mobilenet v2, no augmentation	84.6–46.1%	62.3% | 10.7	84.5–44.8	62.0 | 10.8	85.0–45.4	62.8 | 10.8

## Data Availability

The data presented in this study are available on request from the corresponding author.

## Nomenclature

Conv1d	Layer in deep neural networks that performs a convolution on a one-dimensional signal.
MaxPool1d	Layer in deep neural networks that performs a pooling operation by selecting the maximum value from the moving window.
Fully connected	Layer in deep neural networks that consists of neurons, each of which process the whole input data.
Leaky ReLU	Activation function used in deep neural networks.
Padding	Parameter used in convolutional layers specifying the amount of zeroed samples added to the start and the end of the processed signal. For example: a padding of 1 means that there is one sample of value zero artificially added on the beginning and at the end of the signal. This operation is conducted in order to mitigate activation-map shrinkage due to the application of convolution.
Stride	Parameter used in convolutional layers specifying the shift distance between subsequent windows of convolutions. For example: a stride of 1 means that the next convolution starts right after the the beginning of the previous one, so the windows will overlap (provided that the kernel size is greater than 1).
ALL	Acute lymphoblastic (or lymphocytic) leukemia.
AML	Acute myeloid (or myelogenous) leukemia.
CML	Chronic myeloid (or myelogenous) leukemia.
CLL	Chronic lymphocytic leukemia
WBC	White blood cells.
